# A structure, process and outcome evaluation of the Geriatric Emergency Department Intervention model of care: a study protocol

**DOI:** 10.1186/s12877-017-0462-z

**Published:** 2017-03-23

**Authors:** Elizabeth Marsden, Andrea Taylor, Marianne Wallis, Alison Craswell, Marc Broadbent, Adrian Barnett, Kim-Huong Nguyen, Julia Crilly, Colleen Johnston, Amanda Glenwright

**Affiliations:** 1 Nambour Emergency Department, Sunshine Coast and Hospital Health Service, Hospital Rd, Nambour, QLD 4560 Australia; 2 School of Nursing, Midwifery and Paramedicine, University of Sunshine Coast, 90 Sippy Downs Drive, Sippy Downs, QLD 4556 Australia; 3 Queensland University of Technology, Brisbane, QLD 4059 Australia; 4 Center for Applied Health Economics, Menzies Health Institute Queensland, Griffith University, Logan, Australia; 5 Menzies Health Institute Queensland, Griffith University, Parklands Drive, Parkwood, QLD 4215 Australia; 6 Department of Emergency Medicine, Gold Coast Health, 1 Hospital Blvd, Southport, QLD 4215 Australia; 7 Central Queensland, Wide Bay, Sunshine Coast PHN, Ground Floor, Mayfield House, 29 The Esplanade, Maroochydore, QLD 4558 Australia

**Keywords:** Geriatric, Emergency medical services, Nurses’ practice patterns, Hospital, Homes for the aged, Delivery of health care, Protocol, Outcomes, Evaluation, Pragmatic paradigm

## Abstract

**Background:**

Emergency departments are chaotic environments in which complex, frail older persons living in the community and residential aged care facilities are sometimes subjected to prolonged emergency department lengths of stay, excessive tests and iatrogenic complications. Given the ageing population, the importance of providing appropriate, quality health care in the emergency department for this cohort is paramount. One possible solution, a nurse-led, physician-championed, emergency department gerontological intervention team, which provides frontload assessment, early collateral communication and appropriate discharge planning, has been developed. The aim of this Geriatric Emergency Department Intervention is to maximise the quality of care for this vulnerable cohort in a cost effective manner.

**Methods:**

The Geriatric Emergency Department Intervention research project consists of three interrelated studies within a program evaluation design. The research comprises of a structure, process and outcome framework to ascertain the overall utility of such a program. The first study is a pre-post comparison of the Geriatric Emergency Department Intervention in the emergency department, comparing the patient-level outcomes before and after service introduction using a quasi-experimental design with historical controls. The second study is a descriptive qualitative study of the structures and processes required for the operation of the Geriatric Emergency Department Intervention and clinician and patient satisfaction with service models. The third study is an economic evaluation of the Geriatric Emergency Department Intervention model of care.

**Discussion:**

There is a paucity of evidence in the literature to support the implementation of nurse-led teams in emergency departments designed to target frail older persons living in the community and residential aged care facilities. This is despite the high economic and patient morbidity and mortality experienced in these vulnerable cohorts. This research project will provide guidance related to the optimal structures and processes required to implement the model of care and the associated cost related outcomes.

**Trial registration:**

Australian New Zealand Clinical Trials Registration Number is 12615001157561. Date of registration 29 October 2015.

## Background

Across the developed and developing world the population is ageing. In Australia it is predicted that, compared to 2005 the proportion of the population over 65 years of age will increase from 13% to 24%, by 2036 [1]. With this increase, the demand on Emergency Department (ED) services by older persons living in the community or in residential aged care facilities (RACF) will increase [1–3]. A systematic review of 27 studies from around the world has demonstrated that the rate of transfer to EDs from RACFs is 0.1–1.5 transfers per RACF bed/year [4].

As a result of this ageing of the population it is imperative that the health care system develop safe models of care (MOC) that span both the community and hospital health care sectors. While the ED is uniquely designed to care for the critically ill or injured, it is often a suboptimal environment for the provision of care to the frail, older person [5, 6]. Prior research shows that older persons who develop an acute illness or who have an acute exacerbation of a chronic condition, have complex medical needs and the doctors and nurses who manage their initial care in the ED often have limited experience in geriatric medicine [7, 8]. Several studies have shown that this situation leads to: under-triage; higher rates of admission; delayed and fragmented care; longer stays in the ED; and unnecessary investigations and invasive interventions [9–19]. For patients from RACFs this is particularly true. During admission to hospital, patients from RACFs are at high risk of complications such as delirium, urosepsis, falls and death [20–22]. Patients from RACFs who develop an acute illness or an exacerbation of a chronic condition also suffer high rates of re-presentation to the ED and subsequent re-admission to hospital [2, 10, 12, 14].

Several studies indicate that a large percentage of frail older persons living in the community or RACFs express a preference for treatment in their home if appropriate [23, 24]. Despite this, approximately 60% of ED presentations lead to hospital admission [25, 26]. In an attempt to keep frail older persons at their place of residence during an acute illness, several MOC have been designed and tested. Broadly, the different MOC are divided into those that: i) provide in-home care or outreach of services from one service to another [12, 24, 27, 28]; ii) provide prioritisation or geriatric focused care in the ED [29–32]; or iii) support or enhance primary care [28, 33].

Interventions that focus on improving care in the ED for frail older persons living in the community or RACFs include, but are not limited to: nurse led discharge-planning [27, 32]; volunteers [31]; comprehensive geriatric assessment units; and care coordination programs [34]. It is unclear which MOC for frail older persons visiting EDs, will result in the best outcomes. Few randomised controlled trials of such interventions in the ED setting have been published [35]. Systematic reviews of studies that focus on these types of interventions have found that for the majority of older persons, ED interventions required further research as there is insufficient evidence to ascertain their impact. Crucially the majority of these studies exclude RACF residents, creating a serious evidence gap for this cohort [12, 30, 35]. Aldeen et al. [32] described an ED based geriatric nurse liaison MOC that shows promising results in decreasing hospital admission and safe discharge planning; however, it was limited to older persons living in the community in the American setting.

This study is part of a larger service evaluation project called the Care Coordination through Emergency Department, Residential Aged Care and Primary Health Collaboration (CEDRiC) project. The CEDRiC MOC has interconnecting elements in both the ED and the RACF to improve care for frail older persons and RACF residents in the ED and optimize primary health care. The aim of this article is to present the research protocol for a study that will evaluate the structures, processes and outcomes of an ED focused MOC aimed at improving care for frail older persons living in the community or RACFs who present to the ED with an acute illness.

## Methods/design

### Study aims

The aims of the Geriatric Emergency Department Intervention (GEDI) MOC evaluative research project are to:

1. Test the efficacy of the GEDI MOC by comparing the outcomes of all people aged 70 years or older who presented to a South East Queensland ED before the implementation of the GEDI MOC with those in the 12 months following implementation. Outcomes measured will be: rates of ED representation within 72 h and 28 days, mortality, ED and hospital LoS, time to referral, time to disposition and hospital admission rates;
2. Describe and explore the structures and processes required for the effective delivery of the GEDI MOC;
3. Conduct an economic evaluation of the GEDI MOC.

### Setting

This study is set on the Sunshine Coast of Queensland, Australia. In Australia, the current percentage of people aged 65 years and over accounts for around 14% of the population [1]. On the Sunshine Coast this percentage is expected to increase to 17% by 2031 [36]. The study will be conducted in the Nambour General Hospital, situated in the Sunshine Coast and Hospital Health Service (SCHHS). Nambour General Hospital is one of four public hospitals in the SCHHS. NGH is the base hospital containing 373 beds and the three secondary hospitals have a combined total of approximately 531 beds. All four hospitals have an ED where both adults and children are treated. The study ED has an annual presentation of 52,410 patients; 18% of whom are over the age of 70 years [36]. Locating the research in the SCHHS will provide evidence of the effectiveness and feasibility of this MOC in a regional setting.

### Intervention

GEDI is a nurse-led, physician-championed innovative MOC that aims to reduce frail older person living in the community or RACF ED length of stay and streamline service delivery by maximising the provision of care during the patient’s ED journey. GEDI clinical nurses (CN) have a minimum of five years post registration experience and specialist expertise and/or education in both emergency nursing and care of the older person. Their role focuses on case-managing frail older persons living in the community or RACF in the ED facilitating rapid access and coordination of care through ED, hospital and community services.

There are 2.4 full time equivalent (FTE) GEDI CNs and a 0.8 FTE Clinical Nurse Consultant (CNC) employed by the ED. They cover the ED from 0700 to 1730 Monday to Friday and 0700 to 1530 on weekends. The GEDI CNC oversees the CN role, providing education, leadership, managerial oversight of the service and clinical support where needed. An ED physician provides medical leadership and together with the CNC they are responsible for research and administration.

In the ED, the GEDI team will provide early, rapid, targeted geriatric assessment of frail older persons living in the community or RACF presenting to the ED and, through careful inter-facility and interdisciplinary, management and planning, aim to prevent unnecessary admissions to hospital. In addition, the GEDI CNs provide a single point of contact for ED and RACF staff who may be having difficulty managing an older person with an acute illness. Table 1 outlines the elements included in the GEDI roles. The importance of the ED clinical experience required for the GEDI role relates to the need for GEDI CNs to optimise communication; multitask within a chaotic, fast-paced clinical environment; and be familiar with the staff, clinical pathways and protocols.

**Table 1:** GEDI team responsibilities

GEDI Clinical Nurse Consultant	GEDI Clinical Nurse	GEDI Physician
Clinical responsibilities		
▪ Collaborative partnership with physician-champion to develop evidenced based guidelines specific to the needs of the geriatric patients and clinicians in the ED	▪ Works collaboratively with all ED staff	▪ Clinical resource and expert in geriatric emergency medicine
▪ Develops and maintains enhanced communication strategies related to ED staff, RACF staff, Nurse Practitioner (NP), community services, paramedic staff and general medical practitioners (GPs).	▪ Enhanced communication by providing a dedicated single point of contact within the ED for RACF staff, NP, community services, paramedics and GPs to obtain support and advice regarding optimal care and management of acutely unwell or injured frail older person or RACF resident	▪ Facilitates interdisciplinary discussions and establishes clinical hospital wide networks
▪ Develops and upskills CNs in use of rapid assessment of frail older persons or RACF residents	▪ Rapid assessment and management of frail older persons or RACF resident including nurse initiated analgesia and investigations	▪ Identify information to be sourced by the GEDI CN relevant to ED medical officers
▪ Develops evidence based clinical care algorithms for common problems experienced by RACF residents within the ED	▪ Provides evidence based clinical care for older persons in the ED in collaboration with the primary nurse	▪ Provide education for emergency staff, promoting informed decision making and best practice
▪ Provision of a consultative service for patient centred care of the frail older person or RACF resident within the ED	▪ Provision of a consultative service for patient centred care of the frail older person or RACF resident within the ED	
▪ Develops systems and upskills CNs to undertake direct referral to Geriatricians and rapid consultation pathways with other medical service streams	▪ Direct referral to Geriatricians and rapid consultation pathways with other medical service streams	
▪ Develops systems to facilitate pre-hospital communication with the RACF, GP, NP and Ambulance service, facilitating appropriate transfer decision making and early arrival triage	▪ Pre-hospital communication with the RACF, GP, NP and Ambulance service, facilitating appropriate transfer decision making and early arrival triage	
	▪ Liaison with hospital acute-care substitution services such as the Hospital in The Home and palliative care services	
Administrative responsibilities		
▪ Collaborative partnership with physician-champion to manage the GEDI team and GEDI CN portfolios	▪ Facilitation of discharge back to place of residence or admission – i.e. appropriate disposition planning	▪ Implement MOC and promote care of the older persons in ED
▪ In collaboration with GEDI Physician and university partners coordinate a program of research focused on the care of older persons in ED	▪ Ensuring discharge summaries are provided to all care providers e.g. GPs, RACF, primary carers, community services following acute ED care to allow seamless transition of care	▪ Create and edit clinical pathways and guidelines for best practice i.e. Delirium management
▪ Coordinate the aged care component of the ED staff development program in consultation with GEDI CNs and ED Nurse Educator	▪ Provide education for ED staff in evidence based care of the frail older person or RACF resident	▪ Leader in change management and lean thinking strategies
▪ Coordinate NP clinical practicum in the ED for NP candidates specialising in care of the frail older person or RACF resident to enhance skill base and knowledge of the ED setting. Skills obtained are translated into practice within the aged care service setting.	▪ Facilitate education/clinical exposure in the ED for NP candidates specialising in care of the frail older person or RACF resident to enhance skill base and knowledge of the ED setting.	▪ Negotiation of resource use in the ED
	▪ Establishment of rapid, direct referral pathways to specialised geriatric and palliative care departments	▪ Champion the GEDI model in strategic planning with senior staff and hospital administration
		▪ Collaborate and maintain research relationships
		▪ Participate in research and program audits
		▪ Administrator of GEDI team and budget
		▪ Collaborate with national and international geriatric focus groups
		▪ Liaise with other program directors/coordinators

### Study design

Pragmatism will provide the philosophical paradigm for this mixed model evaluation research. This philosophical paradigm allows the researcher to focus on “what works” and provides solutions for problems utilising methods that best meet their needs and purpose [37]. The design will be underpinned by Donabedian’s structure, process and outcome evaluation of healthcare framework [38].

The three studies included in this protocol will aim to answer the following evaluative research questions:

1. What is the effect of the introduction of the GEDI MOC on the outcomes of frail older persons living in the community or RACF who present to ED? (Outcomes include: rates of ED representation within 72 h and 28 days, mortality, ED and hospital LoS, time to referral and hospital admission rates).
2. What structures and processes are required to deliver the GEDI MOC?
  Specifically:
  - What are the experiences of staff in regards to the processes of the GEDI MOC?
  - What processes pertaining to the GEDI MOC have affected the day to day operation of the ED, and how?
  - What are the residents’ and carers’ experiences of the GEDI MOC?
3. What is the effect of the GEDI MOC on hospital and health service delivery costs?
  Specifically:
  - What is the effect of the introduction of the GEDI MOC on per patient cost associated with ED and hospitalisation?
  - What is the effect of the introduction of the GEDI MOC on preventing the costs associated with avoidable inpatient admissions?

#### Study 1: Pre-post comparison of GEDI MOC in the ED

Study one is a quasi-experiment comparing outcomes for patients seen by the GEDI team to historical controls. As with all quasi-experiments, the disadvantage lies in the internal validity and risk of bias and this experiment will predominantly be affected by selection and historical control bias. Further to the selection bias and given the time difference between the pre and post intervention groups, events within the ED may influence the observed effects and lead to historical bias. Throughout the intervention a record will be made of any process or system changes that occurred within the ED during the GEDI team’s implementation [39]. These will be incorporated into the analysis.

##### Samples

The study sample comprises frail older persons living in the community or RACF presenting to Nambour General Hospital ED during two time-periods:

(i) Time 1: 1^st^ January to 31^st^ December 2012: prior to development of any aspect of GEDI MOC, and
(ii) Time 2: GEDI MOC implementation: 1^st^ September 2015 to 31^st^ August 2016

The gap between these two time-periods was when the GEDI MOC was developing, partially staffed and supported and was, therefore, not operating at an optimal level.

##### Data collection

Data extraction will occur retrospectively via medical records audit of coded state based Health Service databases, namely Emergency Department Information Service (EDIS; Healthcare Group, CSC)® and Hospital Based Corporate Information System (HBCIS; iSoft). SCHHS Health Statistics unit provides linking of the EDIS, HBCIS and financial data providing raw data on all presentations to the ED and those admitted to hospital. The raw data will subsequently be manually cleaned to identify RACF residents based on their home addresses. This is important as RACFs often have independent living community residents at their facilities who needed to be excluded from this cohort. Independent variables that will be used to describe the sample and used to build multivariable models for outcomes are:

- Demographics - Age, Sex, postcode
- Date and time of presentation
- Clinical diagnosis – reason for presentation and ICD-10 code/category
- Seen by GEDI team – Yes/No
- Australasian Triage Score [40]
- Adult Deterioration Detection System Score [41]
- Next of Kin/Substitute decision maker/Enduring Power of Attorney details
- Time to referral from ED arrival
- Time to disposition from ED arrival

Outcome variables collected will be:

- Disposition - discharged home, moved to short stay area, admitted, died
- ED LoS – in minutes
- Hospital LoS – in days
- All cause in-hospital mortality within 30 days of ED presentation
- ED Re-presentations in 72 h and 28 days
- Time to ED re-presentation within 28 days

##### Data analysis

Descriptive statistics will be used to describe the intervention and control groups including frequencies, percentages, appropriate measures of central tendency and distribution. We will use survival analysis to jointly model length of stay and disposition, with the four destinations as competing risks. We will use survival analysis for ED representations with out-of-hospital mortality as a competing risk. All models will adjust for the patient level factors of gender, age, triage score, season, day of the week and time of presentation. Pre-post designs are vulnerable to confounding by other changes over time that may be attributed to the intervention [39]. To control for this, we will include a linear trend (based on date) in all models to account for gradual changes that are not captured in the individual variables, e.g., experience of healthcare workforce. We will also adjust for season using a sinusoid with an annual cycle to control for the winter peak in morbidity [42]. The survival analyses will use Cox models and we will check the proportional hazards assumption. The models’ residuals will be checked for outliers and correlation over time. We will calculate Cook’s influential statistic and examine relatively large outliers. We will calculate the variance inflation factor and remove variables with a score above five on the basis that they are co-linear. The key outcome will be the mean effect of the intervention together with 95% confidence intervals. The results will be reported using the Strengthening the Reporting of Observational Studies in Epidemiology (STROBE) guidelines [43].

#### Study 2: Structure and process evaluation of GEDI

This qualitative phase of the research aims to examine the structures and processes required for effective implementation of a GEDI MOC and staff and patient experience of the model [38, 44]. The structures and processes required for the GEDI MOC evaluation were adapted from Irvine et al.’s (1998) nursing role effectiveness model [45]. Structural elements required to operate GEDI include organisational, patient and staff resources. Process elements include role and relationship requirements that enable the GEDI MOC to operate, such as team communication, nursing interventions and case management.

The focus is on frail older persons living in the community or RACF with acute and chronic medical conditions, their family and the staff interacting with GEDI MOC.

##### Participants

There are two participant groups:

1. Nursing and Medical staff - NGH ED (*n* = 20). Participants in this group will have worked with the GEDI team in the ED.
2. Frail older persons living in the community or RACF who presented to the ED and were seen by the GEDI team and/or their family member (*n* = 10). Participants in this group will have had personal experience or family member experience of their relative being cared for by the GEDI team.

Participants will be recruited via the following methods:

- Emails will be sent to all ED staff via the CNC/Nurse Unit Manager and Medical Director of the ED. A member of the research team will attend staff meetings. Interested staff will then be provided with an information sheet and consent form and an interview arranged at a time and place suitable to the participant.
- Older persons, RACF residents and/or their attending family members will be given an information sheet, consent form and reply paid envelope while in the ED. This group will be sampled via purposeful sampling to obtain information rich cases who have experienced the GEDI MOC. Consent will be obtained in writing at the time of the interview.

##### Data collection

Semi-structured, audio-recorded, individual interviews will be undertaken. Participants will be asked to respond to semi-structured questions framed around the a priori system of catagorising qualitative data as proposed by Bogdan and Biklen [46]. These a priori categories will be delineated by the structures and processes required for the GEDI MOC evaluation, adapted from Irvine et al.’s (1998) nursing role effectiveness model [45]. Recorded interviews will be transcribed verbatim prior to analysis.

##### Data analysis

A priori categorisation ensures the analysts code the interviews relevant to the research questions during the analysis [47]. For each section of qualitative data relating to the relevant a priori category, the transcripts of interviews are read and re-read with an initial label then assigned to sections of text. The use of a priori labels provides a separate accounting scheme for the analysis of qualitative data by limiting the analysis to focus on the structures and processes experienced by participants relevant to the GEDI MOC. Development of categories by assigning textual data to emerging categories, enable comparison and discussion with the research team [48]. NVivo 10 will be used to manage the transcribed interview data.

#### Study 3: Health economics

Individual patient data will be collected from the Hospital Based Corporate Information System (HBCIS) for Nambour General Hospital. HBCIS is a patient record system for hospital administration that includes information such as patient details, ED presentation complaint, diagnosis, length of stay, bed and menu assignment, discharge status and costs associated with ED episode and inpatient episode of care (if the patient was admitted for inpatient care). For the economic analysis, data will be collected from the two periods in time pre and post GEDI MOC implementation. The historical data during the period of 1^st^ January 2012 – 31^st^ December 2012. The experimental data will be collected for the period of 1^st^ September 2015 to 31^st^ August 2016. While de-identified, the costing data contains sufficient information on hospital resource utilisation such as triage category, length of stay, and treatment received as well as discharge status.

##### Data analysis

A multivariate cost function will be estimated to determine average cost per patient, for before- and after-GEDI MOC scenarios. The function takes into account important determinants of individual cost such as age, severity, length of stay and procedures received while in the ED. In the cost effectiveness analysis, we will estimate the average difference in number of admissions for the before- and after-GEDI scenarios. The number of avoidable admissions will be converted to cost savings using the results of the cost analysis above, and compared against the implementation cost for the GEDI MOC. If the cost savings is larger than the implementation cost, the GEDI MOC can be viewed as cost effective.

## Discussion

GEDI is an innovative MOC that provides a single point of contact in the ED for RACF staff, primary health services and secondary health services. The primary focus is to deliver high quality care for frail older persons living in the community or RACF through seamless continuity of care as they transition through the ED. Secondly, the GEDI MOC aims to facilitate community care strategies and thus acts as a hospital substitution model.

Complex, multifocal interventions, such as the GEDI MOC, are frequently implemented without rigorous evaluative research. This study will provide not only a description of the structures and processes required for successful implementation of the MOC but will also provide older person and cost related outcomes.

## Abbreviations

CEDRiC: Care coordination through emergency department, residential aged care and primary health collaboration
CN: Clinical nurse
CNC: Clinical nurse consultant
ED: Emergency department
EDIS: Emergency department information service
FTE: Full time equivalent
GEDI: Geriatric emergency department intervention
GP: General practitioner
HBCIS: Hospital based corporate information system
LoS: Length of stay
MOC: Model of care
NGH: Nambour general hospital
RACF: Residential aged care facility
SCHHS: Sunshine coast and hospital health service
